# A cross-sectional study of the health status of Swiss primary care physicians

**DOI:** 10.1038/s41598-021-02952-2

**Published:** 2021-12-06

**Authors:** Paul Sebo, Thierry Favrod-Coune, Liv Mahler, Amir Moussa, Christine Cohidon, Barbara Broers

**Affiliations:** 1 Institute for Family Medicine and Paediatrics, University of Geneva, Geneva, Switzerland; 2 Primary Care Division, Geneva University Hospital, Geneva, Switzerland; 3 Department of Family Medicine, Center for Primary Care and Public Health (Unisanté), University of Lausanne, Lausanne, Switzerland

**Keywords:** Diseases, Medical research

## Abstract

There is limited data on the general health of primary care physicians (PCPs). We aimed to assess the physical and psychological health of Swiss PCPs. We selected a random sample of 1000 PCPs in Western Switzerland. They were asked about their self-rated health status, all medical conditions experienced in the past five years, and the number of days they were hospitalized and off work in 2019. They were also asked whether they had their own general practitioner (GP) and seen a psychiatrist/psychologist in the past 12 months. A total of 503 PCPs were included in the study (women = 51%, GPs = 67%, pediatricians = 19%, gynecologists = 14%). Ninety-four percent considered themselves in good or very good health. In the past five years, PCPs suffered mostly from depression/anxiety (21%), burnout (21%), dyslipidemia (19%) and hypertension (17%). Male and older PCPs had more often cardiovascular disorders, younger PCPs and GPs had more often psychiatric disorders. They were 9% to have been hospitalized (15% for PCPs over 60) and 20% to have been off work (32% for PCPs under 45). Only 47% had their own GP (37% for GPs). They were 16% (mostly female and younger PCPs) to have consulted a psychiatrist/psychologist. In conclusion, although PCPs considered themselves to be in good health, a substantial proportion suffered from a medical condition, mainly psychiatric (depression or burnout) and/or cardiovascular disorders, or were recently hospitalized or off work. Only half had a GP for themselves. These results may be useful for implementing specific health strategies targeting PCPs.

## Introduction

Several studies explored the health status of physicians, including primary care physicians (PCPs), because of the proven link between their health and the quality of patient care^1–3^. These studies focused primarily on psychiatric illness^4–11^ and addiction^12–19^. We lack data regarding their physical health.

The psychological health status of physicians, a growing source of concern, is linked to multiple causes (economic constraints, clinical or administrative workload, high-stress load, isolation, lack of recognition and job-related dissatisfaction)^2,4–6^. The prevalence of depression, addictions, burnout and suicides is particularly high in this population. For example, a systematic review showed that physicians in the UK had a prevalence of psychiatric disorders between 17 and 52%^7^, and according to a nationwide US study 46% of physicians reported at least one symptom of burnout^8^.

PCPs appear to be particularly at risk. A study conducted in the UK found that 30% of PCPs suffered from mental distress^9^. A systematic review found that the prevalence of burnout among American PCPs ranged from 14 to 60%^10^. A third study conducted in 12 European countries and using the *Maslach Burnout Inventory* found that 43% of PCPs scored high for emotional exhaustion, 35% for depersonalization, 32% for personal accomplishment, and 12% for all three dimensions^11^.

Due to limited data on the overall health status of PCPs, we launched a questionnaire-based survey in 2020 to assess the physical and psychological health of Swiss PCPs (including pediatricians and gynecologists).

## Methods

### Study site and study population

This cross-sectional study was conducted in November and December 2020 in Western Switzerland (seven cantons: Geneva, Vaud, Neuchatel, Valais, Fribourg, Jura and Bern). We selected a non-stratified random sample of 1000 physicians from the list of 2455 PCPs practicing in Western Switzerland (men: 53.4%; general practitioners (GPs): 69.0%, pediatricians: 17.5%, gynecologists: 13.5%). We used the database of the Swiss Medical Association (FMH, *Foederatio Medicorum Helveticorum*) which lists physicians practicing in Switzerland. Selected physicians were invited to participate by post. Reminder messages (one per physician) were sent to non-responders. Those who did not practice at the time of the study were excluded from the study.

### Data collection

PCPs were asked about socio-demographic characteristics: gender, age group, marital status, medical specialty (general internal medicine, pediatrician, gynecologist), type of practice (solo, duo, group practice, other), mean number of half-days worked per week, number of working years in private practice, and mean number of hours worked per week for clinical and administrative work, respectively. They were also asked about their health status: self-rated health status (excellent, very good, good, moderate, poor), has his/her own GP [Y/N], has seen his/her GP or a psychiatrist/psychologist in the past twelve months [Y/N], number of days hospitalized and number of days off work due to illness and injury in 2019. All medical conditions experienced during the past five years, and smoking status, were recorded according to a list prepared by the research team (Box 1). Finally, they were asked to report all medications taken at the time of the study. Only drugs taken regularly (i.e., at least once a week) were considered.

The location of practice was categorized into urban, semi-urban and rural using the postal code. We referred to the typology of municipalities (*communes*) established by the Swiss Federal Statistical Office (FSO). We also created the variable 'modifiable cardiovascular risk factor', ranging from zero to five according to the number of risk factors among the following: hypertension, diabetes, dyslipidemia, obesity, and smoking.

Participants who preferred to complete an online version of the questionnaire were invited to log in via a hyperlink and complete the questionnaire after entering their participation code. There was no financial compensation for participation. The questionnaire was pretested by five PCPs to identify difficulties in responding to the questions, and adapted after their suggestions. The web-based questionnaire was as similar as possible to the paper version, including regarding the text formatting. All methods were carried out in accordance with relevant guidelines and regulations.

### Statistical analyses and sample size

We used frequency tables to summarize categorical variables and medians and inter-quartile ranges (IQRs) to summarize discrete numerical data. We compared health status in subgroups of PCPs using chi-squared tests and univariable logistic regression for unadjusted analysis, and multivariable logistic regression for adjusted analysis. We only analyzed self-reported medical conditions that were present in at least 10% of study participants. For the multivariable analysis, we created two models. For model #1, we included seven socio-demographic factors (gender, age group, medical specialty, type of practice, location of practice, hours worked, and civil status), whether or not they were associated with the dependent variable in univariable analysis. We decided to include these variables because we theoretically considered them important potential confounders. For model #2, we used a non-automatic backward stepwise procedure to remove any covariates associated with a *p* value ≥ 0.1.

The sample size was calculated to estimate the proportion of 50% (= the proportion with the largest sample size), with a 95% confidence interval width of 0.10 (10%) around the estimate. The minimal sample size required for the study was 385. Given the expected 40% participation rate, 1000 physicians were invited to participate.

The statistical significance was set at a *p* value of ≤ 0.05. All statistical analyses were performed with STATA 15.1 (College Station, USA).

### Confidentiality and ethical approval

All data were collected in an anonymous manner. Informed consent was obtained from all participants. The research protocol was approved by the Research Ethics Committee of Geneva University (project-ID: 2019-01850).

## Results

Of the 1000 PCPs contacted, 510 agreed to participate in the study (participation rate: 51%). Seven physicians were excluded because they did not practice at the time of the study. Table 1 summarizes PCPs’ socio-demographic characteristics. Fifty-one percent were women, 72% were < 60 years, two-thirds were GPs. Compared to the initial list of 2455 PCPs practicing in Western Switzerland, the study sample was comparable regarding gender and medical specialty (men = 53.4% vs. 48.7% in our study; GPs = 69.0% vs. 66.9%, pediatricians = 17.5% vs. 19.1%, gynecologists = 13.5% vs. 14.0).

**Table 1:** Primary care physicians’ sociodemographic characteristics (N = 503).

Characteristic	N (%)	Median (IQR)
**Gender (N = 503)**		
Female	258 (51.3)	
Male	245 (48.7)	
**Age group (N = 503)**		
< 45 years	139 (27.6)	
45–60 years	225 (44.8)	
> 60 years	139 (27.6)	
**Civil status (N = 501)**		
Single	69 (13.8)	
Married	352 (70.2)	
Divorced or separated	74 (14.8)	
Widowed	6 (1.2)	
**Medical specialty (N = 498)**		
General internal medicine	333 (66.9)	
Pediatric	95 (19.1)	
Gynecology	70 (14.0)	
**Type of practice (N = 502)**		
Solo	171 (34.1)	
Duo	105 (20.9)	
Group	201 (40.0)	
Other^a^	25 (5.0)	
**Location of practice (N = 503)**		
Urban	210 (41.7)	
Semi-urban	191 (38.0)	
Rural	102 (20.3)	
**Number of half-days worked per week (N = 500)**		8 (2)
< 7	122 (24.4)	
7–8	186 (37.2)	
> 8	192 (38.4)	
**Number of working years in private practice (N = 487)**		13 (16)
**Average number of hours per week for clinical work (N = 492)**		32 (15)
**Average number of hours per week for administrative work (N = 493)**		6 (6)

^a^Outpatient hospital service (n = 14), clinic (n = 7), nursing home (n = 4).

Table 2 shows PCPs’ medical characteristics, overall and stratified by gender, age group and medical specialty. Fifty-seven percent of PCPs reported excellent or very good health. About half had their own GP, and of these, half had consulted him/her in the past 12 months. Fewer (16%) had consulted a psychiatrist or psychologist during the same period. Nine percent of PCPs were hospitalized in 2019 (median: 4 days) and 20% were off work during the same year (median: 5 days). PCPs were more frequently hospitalized for accidents than for illness (7% vs. 3% in 2019). They were more likely to be off work for illness than for accident (17% vs. 4% in 2019) but the number of days off work was on average higher for accident than for illness (12 days vs. 5 days). PCPs hospitalized in 2019 and those off work during the same year were more likely to have their own GP than others (data not shown in the table). The figures were 74% vs. 26% (*p* value < 0.001) for hospitalizations overall, 85% vs. 15% (*p* value = 0.01) for hospitalizations for illness, and 69% vs. 31% (*p* value = 0.01) for hospitalizations for accident. The figures were 64% vs. 37% (*p* value = 0.001) for off work overall, 61% vs. 39% (*p* value = 0.01) for off work for illness, and 83% vs. 17% (*p* value = 0.002) for off work for accident.

**Table 2:** Primary care physicians’ medical characteristics stratified by gender, age group and medical specialty (N = 503).

Characteristic	Overall N (%)	Men N (%)	Women N (%)	*p* value^a^	< 45 years N (%)	45–60 years N (%)	> 60 years N (%)	*p* value^a^	General internal medicine N (%)	Pediatric or Gynecology N (%)	*p* value^a^
**General health status (N = 499)**				0.56				0.37			0.15
Excellent/very good	282 (56.5)	143 (58.9)	139 (54.3)		71 (51.1)	126 (56.5)	85 (62.0)		178 (53.5)	102 (62.6)	
Good	187 (37.5)	87 (35.8)	100 (39.1)		58 (41.7)	82 (36.8)	47 (34.3)		133 (39.9)	53 (32.5)	
Moderate/poor	30 (6.0)	13 (5.3)	17 (6.6)		10 (7.2)	15 (6.7)	5 (3.7)		22 (6.6)	8 (4.9)	
**Has his/her own GP (N = 500)**	237 (47.4)	102 (47.2)	135 (52.3)	0.02	76 (54.7)	98 (43.8)	63 (46.0)	0.12	124 (37.4)	111 (67.3)	< 0.001
**Has seen his/her GP in the past 12 months (N = 236)**	126 (53.4)	56 (55.5)	70 (51.9)	0.58	34 (44.7)	50 (51.6)	42 (66.7)	0.03	65 (52.9)	60 (54.1)	0.85
**Has seen a psychiatrist or a psychologist in the past 12 months (N = 500)**	79 (15.8)	23 (9.5)	56 (21.8)	< 0.001	31 (22.3)	43 (19.3)	5 (3.6)	< 0.001	49 (14.8)	30 (18.2)	0.33
**Has been hospitalized in 2019 (N = 497)**	43 (8.7)^b^	22 (9.1)	21 (8.2)	0.74	12 (8.6)	11 (5.0)	20 (14.6)	0.01	23 (7.0)	19 (11.6)	0.08
**Has been off work due to illness in 2019 (N = 496)**	97 (19.6)^c^	42 (17.4)	55 (21.7)	0.23	44 (31.7)	35 (15.9)	18 (13.1)	< 0.001	63 (19.2)	34 (20.7)	0.68

^a^Chi-square tests.

^b^Median 4 days (IQR 5); hospitalization for illness: N = 13 (2.6%), median 4 days (IQR 2); hospitalization for accident: N = 33 (6.6%), median 5 days (IQR 4).

^c^Median 5 days (IQR 13); off work due to illness: N = 83 (16.7%), median 5 days (IQR 12); off work due to accident: N = 18 (3.6%), median 11.5 days (IQR 28).

Compared to men, women were more likely to have their own GP and to consult a psychiatrist or psychologist. Compared to younger physicians, their older counterparts more frequently visited their GP but less frequently a psychiatrist or psychologist. They were more likely to be hospitalized but less likely to be off work. Finally, pediatricians/gynecologists were more likely to have their own GP than GPs. These differences were also statistically significant after adjustment for gender, age group, and medical specialty, except for the association between gender and having their own GP (Supplementary Table 1).

Overall, 54% of PCPs experienced at least one medical condition in the past five years (Table 3), mainly depression and/or anxiety (21%), burnout (21%), dyslipidemia (19%), and hypertension (17%). Sixty-one percent of PCPs had no modifiable cardiovascular risk factors. Twenty-seven percent had one risk factor, 9% had two, 2% had three, and 1% had four. No PCP had all five risk factors.

**Table 3:** Primary care physicians’ medical conditions within the last 5 years (N = 503).

Item	N (%)^a^	Median (IQR)	Min–max
**Medical conditions reported by at least 1% of participants**			
Depression and/or anxiety	104 (20.7)		
Burnout	104 (20.7)		
Dyslipidemia	96 (19.1)		
Hypertension	84 (16.7)		
Asthma	38 (7.6)		
Obesity	35 (7.0)		
Ischemic heart disease	15 (3.0)		
Diabetes	11 (2.2)		
Prostate cancer	8 (1.6)		
Chronic obstructive pulmonary disease (COPD)	5 (1.0)		
At least one medical condition	268 (53.3)	1 (2)	0–6
**All medical conditions assessed in the study**^**b**^			
At least one medical condition	270 (53.7)	1 (2)	0–6
**Number of cardiovascular risk factors**^**c**^			
0	306 (60.8)		
1	135 (26.8)		
2	47 (9.4)		
3	10 (2.0)		
4	5 (1.0)		

^a^No missing data.

^b^Medical conditions: hypertension, diabetes, dyslipidemia, obesity, ischemic heart disease, heart failure, stroke or TIA, peripheral arterial disease, asthma, COPD, colon or rectal cancer, prostate cancer, breast cancer, lung cancer, other cancer, depression and/or anxiety, bipolar disorder, and burnout.

^c^Cardiovascular risk factors: hypertension, diabetes, dyslipidemia, obesity, and smoking.

Table 4 (univariable analysis) and Table 5 and Supplementary Table 2 (multivariable analysis: model#1 and #2, respectively) present the level of association between the medical conditions reported by at least 10% of PCPs and their sociodemographic characteristics. In multivariable analysis, men reported dyslipidemia and hypertension more frequently than women. Older PCPs reported depression/anxiety and burnout less often, but dyslipidemia and hypertension more often than their younger counterparts. GPs tended to suffer burnout more frequently than pediatricians and gynecologists. PCPs practicing in rural areas were more likely to have hypertension than their ‘urban’ colleagues, and they also tended to suffer depression/anxiety more frequently (the difference between the two groups reached statistical significance in model #2, but just not in model #1). Married PCPs reported depression/anxiety less often than non-married PCPs. The results of the two models were similar.

**Table 4:** Unadjusted associations between medical conditions present in at least 10% of study participants and sociodemographic characteristics.

Characteristics	Depression and/or anxiety	*p* value^a^	Burnout	*p* value^a^	Dyslipidemia	*p* value^a^	Hypertension	*p* value^a^	At least one cardio-vascular risk factor^b^	*p* value^a^
	OR (95% CI)		OR (95% CI)		OR (95% CI)		OR (95% CI)		OR (95% CI)	
Men (vs. women)	0.8 (0.5–1.2)	0.31	0.9 (0.6–1.4)	0.56	2.1 (1.3–3.3)	0.001	3.9 (2.3–6.6)	< 0.001	2.7 (1.9–3.9)	< 0.001
≥ 60 years (vs. < 60)	0.4 (0.3–0.8)	0.01	0.4 (0.2–0.7)	0.002	3.1 (2.0–4.9)	< 0.001	4.5 (2.8–7.4)	< 0.001	3.0 (2.0–4.5)	< 0.001
General internal medicine (vs. pediatric or gynecology)	1.6 (1.0–2.7)	0.05	1.7 (1.1–2.9)	0.03	1.2 (0.7–2.0)	0.45	1.7 (1.0–2.9)	0.06	1.3 (0.9–1.9)	0.20
Group practice (vs. solo or duo)	1.6 (1.1–2.5)	0.03	1.3 (0.8–2.0)	0.25	0.6 (0.3–0.9)	0.01	0.6 (0.3–0.9)	0.02	0.6 (0.4–0.9)	0.01
Rural (vs. urban or semi-urban)	1.8 (1.1–3.0)	0.02	1.6 (1.0–2.7)	0.06	1.1 (0.7–1.9)	0.67	1.9 (1.1–3.2)	0.02	1.3 (0.8–2.0)	0.25
> 8 half-days worked per week (vs. ≤ 8)	1.0 (0.6–1.6)	0.99	1.2 (0.8–1.9)	0.36	1.1 (0.7–1.7)	0.72	1.4 (0.9–2.3)	0.13	1.4 (0.9–2.0)	0.10
Civil status: other^c^ (vs. married)	1.7 (1.1–2.7)	0.02	1.4 (0.9–2.3)	0.13	0.9 (0.6–1.5)	0.70	1.0 (0.6–1.7)	0.93	1.2 (0.8–1.7)	0.46

^a^Univariable logistic regression.

^b^Cardiovascular risk factors: hypertension, diabetes, dyslipidemia, obesity and smoking.

^c^Single, divorced, separated or widowed.

**Table 5:** Adjusted associations between medical conditions present in at least 10% of study participants and sociodemographic characteristics.

Characteristics	Depression and/or anxiety	*p* value^a^	Burnout	*p* value^a^	Dyslipidemia	*p* value^a^	Hypertension	*p* value^a^	At least one cardio-vascular risk factor^b^	*p* value^a^
	OR (95% CI)		OR (95% CI)		OR (95% CI)		OR (95% CI)		OR (95% CI)	
Men (vs. women)	0.9 (0.5–1.5)	0.67	0.9 (0.6–1.5)	0.78	1.7 (1.0–2.9)	0.05	2.9 (1.6–5.3)	0.001	2.4 (1.5–3.6)	< 0.001
≥ 60 years (vs. < 60)	0.5 (0.3–0.9)	0.02	0.4 (0.2–0.8)	0.01	2.4 (1.5–4.0)	0.001	3.2 (1.9–5.5)	< 0.001	2.2 (1.4–3.4)	< 0.001
General internal medicine (vs. pediatric or gynecology	1.5 (0.9–2.6)	0.11	1.7 (1.0–2.9)	0.05	1.1 (0.7–1.9)	0.71	1.2 (0.7–2.3)	0.49	1.1 (0.7–1.7)	0.73
Group practice (vs. solo or duo)	1.3 (0.8–2.1)	0.21	1.0 (0.7–1.7)	0.88	0.6 (0.4–1.0)	0.06	0.6 (0.3–1.0)	0.07	0.7 (0.5–1.0)	0.06
Rural (vs. urban or semi-urban)	1.6 (1.0–2.8)	0.06	1.5 (0.9–2.5)	0.15	1.1 (0.6–2.0)	0.72	2.1 (1.2–3.9)	0.01	1.3 (0.8–2.1)	0.29
> 8 half-days worked per week (vs. ≤ 8)	1.0 (0.6–1.7)	0.96	1.2 (0.7–2.0)	0.49	0.9 (0.5–1.5)	0.69	1.0 (0.6–1.7)	0.98	1.0 (0.7–1.5)	0.99
Civil status: other^c^ (vs. married)	1.7 (1.1–2.8)	0.02	1.5 (0.9–2.3)	0.12	1.0 (0.6–1.7)	0.96	1.3 (0.7–2.2)	0.43	1.3 (0.9–2.0)	0.19

^a^Multivariable logistic regression (adjusted for all factors listed in the table).

^b^Cardiovascular risk factors: hypertension, diabetes, dyslipidemia, obesity and smoking.

^c^Single, divorced, separated or widowed.

Finally, the three main medications used by PCPs (Table 6) were paracetamol (16% of physicians), proton pump inhibitors (14%) and anti-inflammatory drugs (13%). More generally, 23% of PCPs regularly took at least one painkiller, 21% at least one cardiovascular drug, and 14% at least one psychotropic drug. The same percentage (14%) reported taking an antidepressant and/or a sleeping pill and/or an anxiolytic.

**Table 6:** Primary care physicians' current medications (N = 503).

Current medication^a^	N (%)^b^
**Pain**	
Paracetamol	80 (16.0)
Nonsteroidal anti-inflammatory drug (NSAID) or COX-2 inhibitor	64 (12.7)
Weak opioid	8 (1.6)
Gabapentin or pregabalin	5 (1.0)
At least one medication used	117 (23.3)
**Cardiovascular disease**	
ACE inhibitor (ACEI) or angiotensin II receptor blocker (ARB)	50 (9.9)
Statin	44 (8.8)
Beta-blocker	32 (6.4)
Platelet anti-aggregant	28 (5.6)
Calcium antagonist	18 (3.6)
Diuretic	16 (3.2)
Anticoagulant	7 (1.4)
At least one medication used	105 (20.9)
**Endocrine or bone disease**	
Calcium and vitamin D	44 (8.8)
Treatment of hypothyroidism	18 (3.6)
Metformin	8 (1.6)
At least one medication used	80 (15.9)
**Psychiatric disease**	
Antidepressant	33 (6.6)
Benzodiazepine	20 (4.0)
Benzodiazepine analog	17 (3.4)
At least one medication used	72 (14.3)
**Respiratory or allergic disease**	
Antihistamine	16 (3.2)
Inhaled corticosteroid	14 (2.8)
LABA, LAMA or SAMA	12 (2.4)
SABA	9 (1.8)
At least one medication used	40 (8.0)
**Other**	
Proton-pump inhibitor (PPI)	70 (13.9)
Treatment of benign prostatic hyperplasia	7 (1.4)
Treatment of hyperuricemia	6 (1.2)
At least one medication used	84 (16.7)

^a^Only medications taken regularly (i.e., at least once a week) and used by at least 1% of study participants are included in the table. Note that for the variable "at least one medication used" all participants' responses were included.

^b^No missing data.

**Box 1:** List of medical conditions from the past five years evaluated in the study.

Hypertension
Diabetes
Dyslipidemia
Obesity
Ischemic heart disease
Heart failure
Stroke or transient ischemic attack (TIA)
Peripheral arterial disease
Asthma
Chronic obstructive pulmonary disease (COPD)
Colon or rectal cancer
Prostate cancer
Breast cancer
Lung cancer
Other cancer (specify)
Depression and/or anxiety
Bipolar disorder
Burnout

## Discussion

### Summary

We found that 94% of PCPs considered themselves in good or very good health, despite 54% having had at least one medical condition in the past five years and only 47% having their own GP (37% for GPs). They were 16% (mostly female and younger PCPs) to have consulted a psychiatrist in the last 12 months, 9% to have been hospitalized in 2019 (mostly PCPs over 60) and 20% to have been off work in 2019 (mostly PCPs under 45). In the past five years, PCPs suffered mostly from depression and/or anxiety, burnout, dyslipidemia and hypertension. Male and older PCPs had more often cardiovascular diseases, whereas younger PCPs had more often depression/anxiety and burnout and GPs had more often burnout.

### Comparison with existing literature

We found that 94% of PCPs considered themselves in good or very good health. There were no significant differences by gender, age, or medical specialty. These results are even better than those of a recent study in France that showed that 80% of GPs considered themselves to be in good or very good health^20^. They are also slightly better than those available for the general population in Western Switzerland (good or very good health status: 87%, data from the 2017 Swiss Health Survey)^21^.

The fact that half of PCPs (and nearly two-thirds of GPs) did not have their own GP is not really surprising considering previously published studies. A US study (2610 internists) found that 50% of respondents did not have a personal physician and 55% had not undergone a clinical examination in the previous three years^22^. Another study conducted among 141 US physicians, the majority of whom were PCPs, found that 63% of them relied on self-diagnosis and self-care^23^, and a French survey (552 physicians in Normandy) showed that less than one in five physicians had an attending doctor other than him/herself^24^. Our finding may be partly explained by a sense of invincibility or omnipotence, or fear of being judged incompetent. Physicians may feel a stigma about admitting they are ill for fear that it will question their competency^2,25,26^. It was shown, for example, that ‘sickness presentism’ was a reality for 80–90% of European physicians, a figure that is much higher than that found in other professions^25^.

Despite very good perceived health, one-fifth of PCPs reported experiencing psychiatric disorders (depression/anxiety or burnout) in the past five years, and one-sixth reported consulting a psychiatrist recently. Younger physicians and GPs appeared to be particularly at risk. The literature indicates that the prevalence of psychiatric disorders, including depression and burnout concerns between 14 and 60% of physicians^2,4–6,9,10,27^, and some of these studies already pointed to the young age and medical specialty (GP) as risk factors for these disorders^7,11,28,29^. It can be hypothesized that younger physicians have less experience and may have fewer internal resources to combat negative emotions. For example, it was shown that there were age differences in the type of defense mechanisms that individuals use, with younger individuals using mature defense mechanisms less often than others^30^. The reasons for GPs' greater susceptibility to psychiatric disorders are unclear. It is likely, however, that factors related to GPs' workstyle and lifestyle are involved, as well as other factors that may vary between medical specialties, such as workload, social recognition and level of emotional involvement in the care relationship with patients. According to the 2017 Swiss Health Survey that used the *Patient Health Questionnaire* (PHQ)-9, the prevalence of depressive symptoms in the last 2 weeks in the general population was 12% in Western Switzerland; still no data are available on 5-year prevalence.

PCPs also suffered from a variety of somatic disorders. Those related to the cardiovascular system (particularly dyslipidemia and hypertension) were the most common. While 19% and 17% reported dyslipidemia and hypertension, respectively, only 7% had obesity and 2% had diabetes. More generally, 39% of physicians had at least one modifiable cardiovascular risk factor. As expected from studies conducted on non-physician populations^31,32^, male and older PCPs were more likely to report cardiovascular disorders. Age is indeed an independent risk factor for cardiovascular disease in adults. It is hypothesized that the age-related increase in oxidative stress results in increased susceptibility to functional and electrical abnormalities that leads to cardiovascular disease^31^. Gender differences in cardiovascular disease are complex to analyze but the causes can be classified into two main groups: common factors and female-specific factors^32^. Women have been shown to be relatively protected from cardiovascular disease during their reproductive lives by sex hormones, a protection that disappears at menopause. It also appears that, although women and men share most of the classic risk factors, the relative weight of these factors is different. For example, hypertension and LDL cholesterol levels appear to have a greater effect in men than in women. There are also gender inequalities in the diagnostic process, with women being investigated less often than men for cardiovascular symptoms^33^. All these differences could explain why women generally have a lower incidence of cardiovascular disease than men, as we found in our study.

PCPs seem to have better somatic health than the general population in Western Switzerland (obesity: 12% in the general population vs. 7% in our study; asthma: 11% vs. 8%; diabetes: 5% vs. 2%; chronic obstructive pulmonary disease (COPD) 3% vs. 1%). The only exception may be hypertension, the prevalence of which was slightly lower in the general population (15% vs. 17% in PCPs), but our study referred to the last 5 years. It should be emphasized that direct comparison between our results and those from population-based studies is limited by the fact that health status is associated with multiple risk factors, such as age and gender, which are not taken into account in this comparison. In addition, although the Swiss Health Survey was based, like our study, on responses to a questionnaire, the definitions used for medical conditions were slightly different. Obesity, defined by a BMI value ≥ 30, was based on self-reported weight without clothing and self-reported height without shoes. Responders were considered to have asthma, respectively COPD, if they reported being diagnosed with asthma, respectively COPD, chronic bronchitis or emphysema, by a physician or health care professional. They were considered diabetic if they reported having diabetes and/or taking medication for diabetes, and were considered hypertensive if they reported having high blood pressure and/or taking medication for high blood pressure.

Nearly one in ten PCPs reported being hospitalized in 2019 and two in ten reported being off work in the same year. Many of these physicians had their own GP. Hospitalizations were more frequently related to accidents and work stoppages to illness. As expected, we found that older PCPs were more frequently hospitalized than their younger colleagues (those over 60 were 2.6 times more likely to be hospitalized than those under 60). More surprisingly, we found the opposite for work stoppages (those under 60 were 1.7 times more likely to be on work stoppage than those over 60).

Our study also looked at the medications commonly used by PCPs. Analgesics (paracetamol and anti-inflammatory drugs) and proton-pump inhibitors were the most frequently used drugs. More generally, 23% of PCPs regularly took at least one painkiller, 21% at least one cardiovascular drug and 14% at least one psychotropic drug. In addition, 14% reported taking an antidepressant and/or a sleeping pill and/or an anxiolytic. Regarding psychotropic medications, the study figures appear to be two times higher than those available for the general population, as 7% of the general population reported use of antidepressants and/or sleeping pills and/or anxiolytics at the time of the study.

Working conditions put many physicians under pressure, with young physicians being particularly affected. Physicians are often reluctant to seek medical help. ReMed was created a few years ago by the Swiss Medical Association to help physicians who are experiencing problems related to their work, their family or who suffer from medical conditions, particularly psychiatric conditions or addictions. They can call ReMed to discuss their problem, in complete confidentiality, and work out a personalized treatment plan^34^. In 2020, ReMed received 170 requests^35^.

## Limitations

Our study had a high response rate and only a few missing data, but it has also some limitations. First, the study sample consisted only of PCPs recruited in Switzerland. However, the results are probably generalizable to many countries at the same socio-economic level as Switzerland and with a comparable health care system, in particular European countries. Second, selection bias, including healthy worker bias, cannot be excluded, but should probably be small because the distribution by gender and medical specialty was relatively similar. Healthy worker bias is related to the fact that individuals who agree to participate in studies are expected to be healthier on average than those who refuse to participate, as they are particularly concerned about their health and are generally predisposed to follow medical advice^36^. In consequence, this can underestimate the morbidity in our study. Third, as with all cross-sectional studies, the various associations found obviously do not imply causality. Finally, the method used to collect the study data (i.e., self-administered questionnaires consisting mainly of simple one-item questions) may result in measurement error. Although the questionnaire was anonymous, some physicians may have tended to underestimate the seriousness of their health problems, especially those with negative connotations, such as psychiatric disorders and obesity. Others may have been tempted to paint a negative picture of their health status in order to alert researchers and/or public authorities to the increasing difficulties encountered by physicians in their daily practice (e.g., more frequent conflicts with health insurance companies and/or increasing administrative work). Well-being scales may also lead a number of respondents to the very low end (i.e., floor effect) or the very high end of the scale (i.e., ceiling effect), making discrimination among these subjects difficult^37^. For logistical reasons we used simple questions to assess physicians' health status and not multiple-item instruments, such as PHQ-9 for screening depression and its severity. Although we used many simple one-item questions, the questionnaire was relatively long to complete. We decided to focus on participation rate and completeness of the questionnaire.

## Conclusion

We found that PCPs considered themselves to be in good health. However, about half suffered from psychiatric (depression or burnout) and/or somatic (mainly cardiovascular) disorders, one-tenth were hospitalized recently and two-tenths off work, and only half had their own GP. We found significant differences according to gender, age or other socio-demographic factors. These results may be useful for implementing specific health strategies targeting PCPs, notably for the importance of having a GP. Future studies are needed to confirm these results in other contexts and to explore other aspects of health, for example those more specifically related to preventive measures.

## Supplementary Information

Supplementary Information.
