# Supplementary material for: A cross-sectional study of the health status of Swiss primary care physicians
Source: Sci Rep. 2021 Dec 6;11:23459. doi: 10.1038/s41598-021-02952-2 (PMC8648724; doi:10.1038/s41598-021-02952-2)
Supplement: Supplementary file 1 — Supplementary Information. [file 41598_2021_2952_MOESM1_ESM.pdf]

Table S1. Adjusted associations between primary care physicians' medical characteristics, and gender, age group and medical specialty (N=503)

| **Characteristic** | **Women (vs. men)** **OR (95%CI)** | **p-value[1]** | **≥ 60 years (vs. < 60)** **OR (95%CI)** | **p-value[1]** | **Pediatric or gynecology (vs. general internal medicine)** **OR (95%CI)** | **p-value[1]** |
|---|---|---|---|---|---|---|
| Has his/her own GP | 1.3 (0.9-1.9) | 0.20 | 1.1 (0.7-1.6) | 0.79 | 3.3 (2.2-4.9) | <0.001 |
| Has seen his/her PCP in the past 12 months | 1.0 (0.6-1.8) | 0.91 | 2.3 (1.2-4.3) | 0.01 | 1.1 (0.6-1.8) | 0.77 |
| Has seen a psychiatrist or a psychologist in the past 12 months | 2.0 (1.2-3.4) | 0.01 | 0.2 (0.1-0.5) | <0.001 | 1.1 (0.7-1.8) | 0.76 |
| Has been hospitalized in 2019 | 1.1 (0.5-2.1) | 0.83 | 2.6 (1.3-5.0) | 0.01 | 1.8 (1.0-3.5) | 0.07 |
| Has been off work due to illness in 2019 | 1.1 (0.7-1.8) | 0.60 | 0.6 (0.3-1.0) | 0.05 | 1.1 (0.7-1.7) | 0.83 |

[1] multivariable logistic regression (adjusted for gender, age group and medical specialty)

Table S2. Adjusted associations between medical conditions present in at least 10% of study participants and sociodemographic characteristics (non-automatic backward stepwise procedure)

| **Characteristics** | **Depression and/or anxiety** | **p-value[1]** | **Burnout** | **p-value[1]** | **Dyslipi-demia** | **p-value[1]** | **Hyper-tension** | **p-value[1]** | **At least one cardio-vascular risk factor[2]** | **p-value[1]** |
|---|---|---|---|---|---|---|---|---|---|---|
| | **OR (95% CI)** | | **OR (95% CI)** | | **OR (95% CI)** | | **OR (95% CI)** | | **OR (95% CI)** | |
| Men (vs. women) | 0.9 (0.6-1.4) | 0.67 | 1.0 (0.6-1.6) | 0.98 | 1.7 (1.0-2.7) | 0.04 | 2.9 (1.6-5.0) | <0.001 | 2.3 (1.5-3.4) | <0.001 |
| ≥ 60 years (vs. < 60) | 0.5 (0.3-0.8) | 0.01 | 0.4 (0.2-0.7) | 0.003 | 2.5 (1.5-4.1) | <0.001 | 3.3 (1.9-5.5) | <0.001 | 2.3 (1.5-3.5) | <0.001 |
| General internal medicine (vs. pediatric or gynecology | 1.6 (1.0-2.7) | 0.07 | 1.8 (1.1-3.0) | 0.02 | NA | NA | NA | NA | NA | NA |
| Group (vs. solo or duo) | NA | NA | NA | NA | 0.6 (0.4-1.0) | 0.07 | 0.6 (0.4-1.0) | 0.07 | 0.7 (0.5-1.0) | 0.08 |
| Rural (vs. urban or semi-urban) | 1.7 (1.0-2.9) | 0.04 | NA | NA | NA | NA | 2.1 (1.2-3.8) | 0.01 | NA | NA |
| > 8 half-days worked per week (vs. ≤ 8) | NA | NA | NA | NA | NA | NA | NA | NA | NA | NA |
| Civil status: other[3] (vs. married) | 1.7 (1.1-2.8) | 0.02 | NA | NA | NA | NA | NA | NA | NA | NA |

[1] multivariable logistic regression (non-automatic backward stepwise procedure: only covariates associated with a p value <0.1 were included in the analysis)

[2] cardiovascular risk factors: hypertension, diabetes, dyslipidemia, obesity and smoking

[3] single, divorced, separated or widowed
